# Sex Determination in Highly Fragmented Human DNA by High-Resolution Melting (HRM) Analysis

**DOI:** 10.1371/journal.pone.0104629

**Published:** 2014-08-06

**Authors:** Brenda A. Álvarez-Sandoval, Linda R. Manzanilla, Rafael Montiel

**Affiliations:** 1 Laboratorio Nacional de Genómica para la Biodiversidad, Unidad de Genómica Avanzada, Centro de Investigación y de Estudios Avanzados del Instituto Politécnico Nacional, Irapuato, Guanajuato, Mexico; 2 Instituto de Investigaciones Antropológicas, Universidad Nacional Autónoma de México, Mexico City, Mexico; University of Florence, Italy

## Abstract

Sex identification in ancient human remains is a common problem especially if the skeletons are sub-adult, incomplete or damaged. In this paper we propose a new method to identify sex, based on real-time PCR amplification of small fragments (61 and 64 bp) of the third exon within the amelogenin gene covering a 3-bp deletion on the AMELX-allele, followed by a High Resolution Melting analysis (HRM). HRM is based on the melting curves of amplified fragments. The amelogenin gene is located on both chromosomes X and Y, showing dimorphism in length. This molecular tool is rapid, sensitive and reduces the risk of contamination from exogenous genetic material when used for ancient DNA studies. The accuracy of the new method described here has been corroborated by using control samples of known sex and by contrasting our results with those obtained with other methods. Our method has proven to be useful even in heavily degraded samples, where other previously published methods failed. Stochastic problems such as the random allele drop-out phenomenon are expected to occur in a less severe form, due to the smaller fragment size to be amplified. Thus, their negative effect could be easier to overcome by a proper experimental design.

## Introduction

Traditionally, sex determination in human remains has been based on the dimorphism between the sexes that is present in the majority of human bones [1]. These studies have been based mainly on cranial and pelvic traits [2]–[15]. Furthermore, other researchers have reported studies based on, hand and foot bones [16], [17], scapula [18], [19], long bones [4], [20]–[26], patellae [27], sternum [28]–[30], fourth rib [28], hyoid bone [30], clavicle [31], meatus acusticus internus characteristics [32], [33] and dentition [34], [35].

Other methods to sex determination have been proposed such as anthropometric measurements of the limbs [36]–[39], hands [40], [41], and from length of index and ring finger, and the index and ring finger ratio [42]–[45]. Nevertheless, it has been reported that 100% of successful sex determinations by osteological measurements only occur when the skeleton is from an adult, it is complete, it is in good condition of preservation, and the morphometric variability in the population to which it belongs is known [46]–[48].

Advances in the field of molecular genetics has provided more sensitive methods for sex determination, such as the polymerase chain reaction (PCR) that allow amplification of single molecules of target DNA to analytical quantities. Biological remains such as hair, bone fragments, or teeth, usually contain some amounts of degraded DNA [48]–[57]; therefore, it is possible to establish an individual sex using genetic test. Recent studies have confirmed that teeth are more refractory to contamination by exogenous DNA than bones, although bones can also be good candidates for analysis under some circumstances [58].

Some proposed methods have been based on the analyses of genetic markers lying in the Y chromosome [59]–[61], or in the use of both X-chromosomal and Y-chromosomal STRs [47]. Furthermore, a new method to sex determination using shotgun sequencing has been reported [62], although it might be too expensive for routine application in a large number of samples. Most of these procedures are not sensitive enough, are time-consuming, expensive, and require an important amount of sample.

Despite the wide list of molecular methods proposed for sex determination, the method based on the amplification of the human amelogenin gene (AMEL) is the most widely used. This gene, originally sequenced by Nakahori *et al* [63], [64], is found on both chromosomes X and Y (GenBank Accession number M55418 for AMELX-allele and M55419 for AMELY-allele). There are sequence and size divergences between them. AMELX-allele has a size of 2872 bp and is located on the Xp22.1–Xp22.3 region of the X-chromosome, while the human AMELY-allele has a size of 3272 bp and is located on the Yp11.2 region of the Y-chromosome. Male individuals have two different AMEL alleles, one located on chromosome X and one on the Y chromosome, while female individuals have two identical AMEL alleles located on chromosome X [63]–[72].

Haas-Rochholz and Weiler [73] reported a total of 19 regions of absolute homology that vary in size from 22 to 80 bp; 5 deletions of 1 to 6 bp located on AmelX-allele; and 5 deletions of 1 to 183 bp located on AmelY-allele thus allowing sex determination by PCR products generated from AMEL-X and AMEL-Y alleles [49], [51], [52], [63], [64], [67], [68], [74]–[76]. The most conventionally used is the 6 bp AMELX-allele specific deletion within intron 1, resulting in 106 and 112 bp PCR products from the X- and Y-alleles, respectively.

However, DNA extracted from less well preserved or older archaeological samples may show higher DNA fragmentation, hampering reproducibility or yielding spurious results [77]. For this reason some researchers have attempted to generate PCR fragments smaller than those described by Sullivan *et al.* [51] but they are still ineffective for heavily degraded samples [73] and can present methodological difficulties because the analysis also involves post-PCR processes. In other instances, the new methods have not been evaluated for degraded or ancient samples [78], [79].

A new strategy developed to sex determination involves the implementation of High Resolution Melting curve analysis (HRM) of PCR products after real-time PCR amplification [80]. This method avoids crossing-over contamination originated from any processing or electrophoretic separations after PCR because the analyses are carried out in the same instrument without exposure of amplicons to the environment (i.e. it does not require any processing or separations after real-time PCR). Furthermore, it allows detection of nonspecific fragments amplified during the PCR step [80]–[83]. Therefore, melting-curve analysis is a convenient method, especially in highly degraded samples, because it is possible to detect a simple mutation in a small amplified DNA fragment, avoiding at the same time further contamination risks. This also allows high-throughput analysis of a large number of samples to be carried out in parallel.

Recently, HRM analysis based on real-time PCR using SYBR Green as a DNA-binding dye has been reported [83]. Differential melting curves were produced by a 3-bp deletion on the X-chromosome corresponding to 70 or 73-bp PCR products. The inefficiency of this dye has also been reported: there is a tendency for it to inhibit PCR, promote mispriming, and the relatively instability of the dye, especially under the alkaline conditions in which it is stored [84]–[86]. Other more important adverse effect of SYBR Green is that it can produce unreliable DNA melting curve data due to the so-called “dye redistribution” phenomenon [87].

Despite the advantages of PCR-based methods, it has been reported that DNA analysis of degraded or ancient samples can present technical difficulties, such as drop-out phenomena observed mainly in the AMELY-allele in male templates with poor amount of DNA [88]–[92]. So far, this situation has been reported only in males with a no-native American origin [93], [94], [95]. According to Kim *et al.,* [92], the most reliable way to avoid the allelic drop-out problem is to replicate PCR results.

We developed a new robust method based on real-time PCR amplification of smaller fragments (61 and 64 bp) of the third exon of the amelogenin gene covering a 3-bp deletion of the AMELX-allele, followed by HRM analysis using EvaGreen as the DNA-binding dye. Our method is rapid, cost-effective, sensitive, and it has proven to be useful in samples showing highly degraded DNA. We present here primers and conditions for the analysis and the results of cross-verification with several controls and methodological comparisons.

## Materials and Methods

### Sampling and Ethics Statement

The study population came from Teopancazco, a multiethnic neighborhood center located in the southern part of Teotihuacan, Central Mexico (200–650 A.D.). Bone and tooth samples were collected in several controlled extensive excavations (1997–2005) and provided by Dr. Linda R. Manzanilla, head of the project “Teotihuacan. Elite and rulership. Excavations in Xalla and Teopancazco” (Last authorizations for the Project: Official letter 401.B (4) 19.2013/36/0579 of the National Institute of Anthropology and History). The controlled extensive excavations, with a 1 meter grid, provided the spatial reference for different activity areas and tridimensional measurement of instruments, raw materials and debris. The sampling process was carried out under strict criteria in order to avoid exogenous contamination. The human burial remains from Teopancazco are kept in the Instituto de Investigaciones Antropológicas (UNAM) under the custody of Dr. Linda R. Manzanilla. The samples are part of the Mexican National Heritage.

A subset of 21 samples (from 21 different individuals buried at Teopancazco) was sent to the aDNA laboratory in LANGEBIO, CINVESTAV, Mexico, in order to be analyzed. Seventeen individuals of unknown sex were analyzed, 14 sub-adults (burials 3, 4, 38, 45, 49, 51, 56, 59, 61, 96, 99, 100, 101 and 110) and 3 adults (burials 46, 89, 108). Moreover, 4 adult individuals of known sex (burials 2, 78, 102 and 105) were used as ancient positive controls. For each individual analyzed, a fragment of bone or a complete teeth, in a good state of preservation (without fissures), was used in the analysis. Preliminary DNA characterization showed a general poor DNA preservation at this archaeological site.

In addition, 2 blood samples donated by the first and third author of this paper [1 male (RM) and 1 female (BAA-S)] were considered as modern positive controls. Both samples were collected under informed consent in order to be considered only as positive controls with no other scientific research objective.

### Genetic analysis

#### 1.- Contamination control

All analyses were carried out under strict conditions for the analysis of ancient DNA, in a dedicated ultraclean room, positively pressurized with filtered and UV-irradiated air, with separation of pre- and post-PCR areas. Laboratory equipments were treated with 30% bleach for DNA contamination removal. The use of disposable filter-plugged pipette tips, tubes, protective cloths, hair covers, laboratory gloves, surgical masks, glasses, and shoes protectors to prevent contaminations was mandatory. Solutions and buffers were irradiated with ultraviolet light. Negative extraction controls and negative PCR controls were always employed.

#### 2.- DNA extraction

Bone and teeth samples were processed in a type II B2 biological security hood, located inside the ultraclean room. The outer surface of the bone or teeth was UV-irradiated during 15 minutes and a dental drill was used to remove 0.1 g of powdered material. Powdered samples were added to 5 ml of extraction buffer (0.5 M Tris-HCl pH 8.0–8.5, 0.5 M EDTA pH 8.0, 0.5% SDS and 0.5 mg Proteinase K) and incubated at 37°C for 24 hrs, followed by phenol/chloroform DNA extraction [96]. Aliquots of crude DNA extracts were analyzed with an *Agilent 2100 Bioanalyzer Expert High Sensitivity DNA Assay* to quantify and assess the quality of extracted DNA.

The fresh blood samples (3 ml) were collected in EDTA vacutainer tubes. Total genomic DNA was isolated using a standard non-organic method and diluted to obtain a working concentration of 2.5 ng/µl. All blood samples were processed in a post-PCR laboratory.

#### 3.- Sex determination by High Resolution Melting Analysis

The HRM analysis was based on the melting temperature (Tm) difference of the amplified AMELX- allele and AMELY-allele fragments (61 bp for the AMELX-allele and 64 bp for the AMELY-allele) of the human amelogenin gene. Fragments were amplified with the *LightCycler 480 Real-Time PCR Instrument* using the *LightCycler 480 High Resolution Melting Master* kit (Roche Applied Science), which contains a saturating fluorescent dye (EvaGreen). The PCR reactions were performed by triplicate (unless indicated) in a total volume of 20 µl containing 2 µl of template DNA (5 ng), 3 mM MgCl_2_, 1X conc. *Lightcycler Master-Mix* [composed by FastStart Taq DNA Polymerase, reaction buffer, dNTP mix (with dUTP instead of dTTP) and High Resolution Melting Dye] and nuclease-free water (QIAGEN), and 0.2 µM of each of the two primers: Amel_F (5′-CCCTTTGAAGTGGTACCAGAG-3′) and Amel_R (5′GGGAATAARGAACAAAATGTCTAC-3′, R = A or G). The primers, designed in our laboratory, flanked a 3-bp GAT insertion on the AMELY-allele located at nucleotide position 1499–1501 on the human Y chromosome (GenBank accession no: M55419).

*The LightCycler 480 Instrument* protocol included a pre-incubation step of 10 min at 95°C. The amplification phase comprised 80 cycles of 15 s, 1 m at 56°C, and 30 s at 72°C. After the PCR step, the High-Resolution Melting analysis was performed measuring the drop of fluorescence signal under the following conditions: 1 m at 95°C, 1 m at 40°C and an increase from 60°C to 90°C at a rate of 1°C/s. The instrument is capable of capturing a large number of fluorescent data points per change in temperature with high precision in order to generate a melting curve chart of sample fluorescence versus temperature using the *LightCycler 480* software *Ver. 1.5* (Roche Applied Science). The analysis also displays the first negative derivate of these sample curves resulting in a new graph where the melting temperature of each sample appears as a characteristic peak. AMELX-allele and AMELY-allele amplicons produced from both modern male and female DNA templates were evaluated as positive controls and three negative controls with no DNA were evaluated in each amplification.

#### 4.- Cross-verification of the High Resolution Melting Analysis by Sanger Sequencing

Cloning of PCR products is useful to assess levels of both contamination and molecular damage in ancient DNA. Also, in our experience, it is not easy to sequence accurately short DNA fragments (<80 bp) by Sanger sequencing, therefore in these cases it is useful to clone the fragments and use vector’s primer-binding sites for sequencing. For these reasons, the AMELX-allele and AMELY-allele amplicons generated from positive controls and from sex-unknown samples were cloned using a TOPO TA Cloning kit (with pCR 2.1-TOPO vector) and TOP10 chemical Competent cells (Invitrogen) following manufacturer’s instructions. A minimum of 30 colonies were selected from each cloned sample and underwent colony PCR using the M13 primers. Then we sequenced 5 clones per sample that contained the transformed vector with the fragment of interest. DNA sequencing was performed in both directions by dideoxy method using the 3730 xl DNA Analyzer (*Applied Biosystems*) at the Genomic Services Unit in Langebio, Cinvestav, Mexico.

#### 5.- Cross-verification of the High Resolution Melting Analysis by Agilent 2100 Bioanalyzer Expert High Sensitivity DNA Assay

A 2 µl aliquot of AMELX-allele and AMELY-allele amplicons generated from positive controls and sex-unknown samples were analyzed by *Agilent 2100 Bioanalyzer Expert High Sensitivity DNA Assay* (the Bioanalyzer assay) with the High Sensitivity DNA Marker 35/10380 bp, using the *Agilent 2100* *Bioanalyzer* (Agilent Technologies Inc.) at the Genomic Services Unit in Langebio, Cinvestav, Mexico.

#### 6.- Cross-verification of the High Resolution Melting Analysis by anthropological data

Human remain samples of 4 adult individuals from Teopancazco (burials 2, 78, 102 and 105) were analyzed by conventional osteological measurements for sex determination at the Institute of Anthropological Research, UNAM, Mexico, under the direction of Linda R. Manzanilla. These samples were used as ancient controls.

## Results

### DNA preservation

Several attempts to determine the sex of these samples with two previously published methods were unsuccessful. These methods targeted amelogenin gene fragments of 106–112 bp [51] and 70–73 bp [83]. This was a first indication of DNA degradation. Consistent with this, the Bioanalyzer assay showed a high DNA fragmentation pattern, with a main distribution of DNA fragments in the range of 40–100 bp, in all ancient samples analyzed (Figure S1). We therefore considered these samples to be heavily degraded and that represented ideal cases to test our HRM method, which targets smaller amelogenine gene fragments.

### Sex determination by High Resolution Melting Analysis

**Figure 1:**
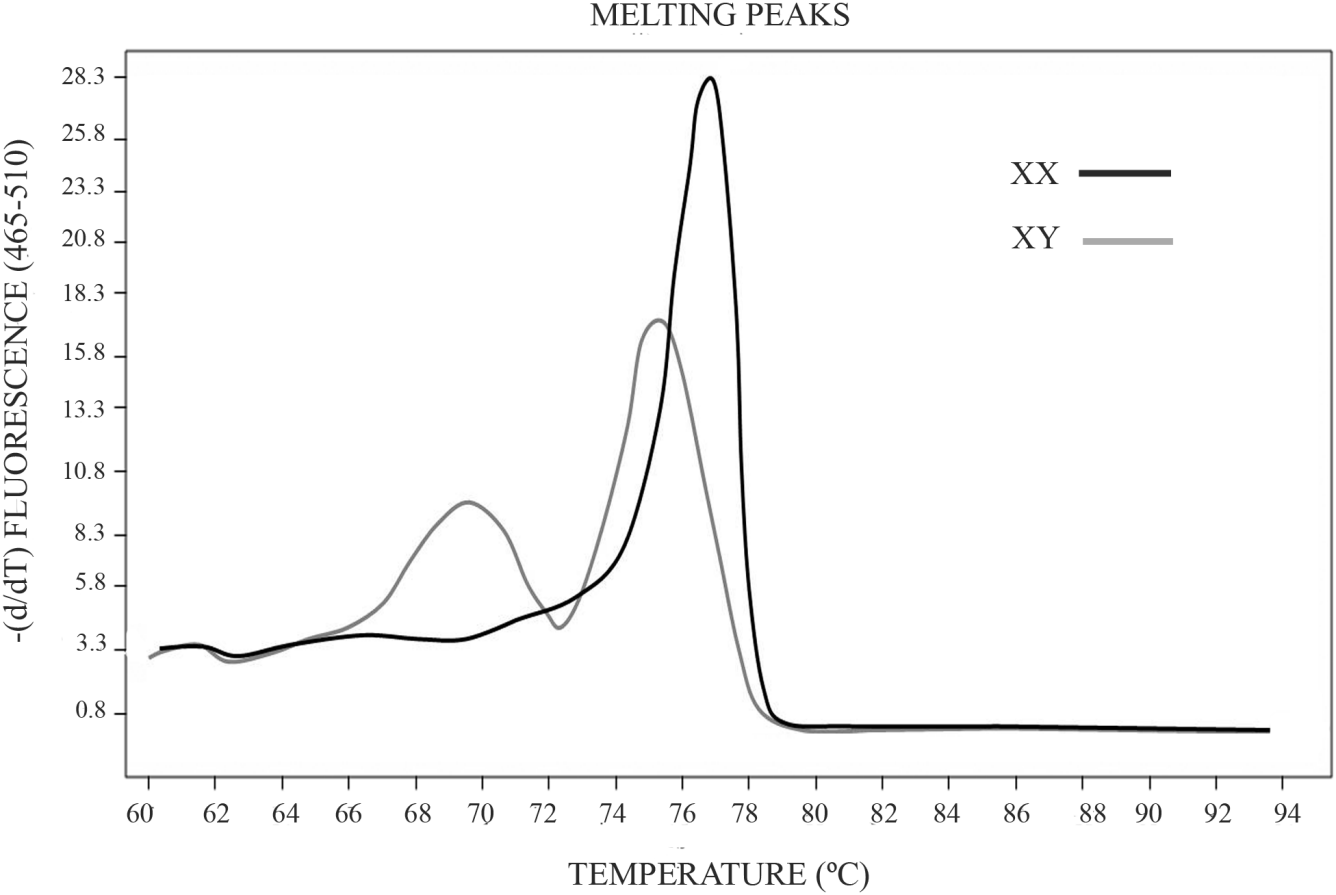
Melting temperature curve profiles produced by male and female samples in a HRM analysis. HRM analysis starts with a PCR amplification of the amelogenin gen. The amplification is in the presence of *EvaGreen* a saturating dye which presents high fluorescence when bound to dsDNA. Amplification is followed by a High Resolution Melting analysis (HRM) in the *LightCycler 480 Real-Time PCR Instrument* (Roche Applied Science). A dissociation curve analysis displays the different melting temperature of products from the X and Y chromosome.

This analysis is based on the melting temperature (Tm) difference of the amplified AMELX-allele and AMELY-allele fragments (61 bp for the AMELX-allele and 64 bp for the AMELY-allele) produced by a 3-bp deletion on the AMELX-allele. After software processing, the curves obtained from male and female individuals showed a clear difference in shape (Figure 1), allowing thus sex identification.

The accuracy of the assay was examined by using a set of 6 control samples of known sex. To assess reproducibility, three replicates of each sample were analyzed. No discrepancy was observed between sex determinations by HRM and the previously known sex status in all control samples and their replicas. Regarding sex-unknown archaeological samples, this method was efficient in all the individuals examined in spite of the observed pattern of DNA degradation. However, for one of the individuals only two replicates were done, because the DNA extract was not enough for the three replicates due to its use in previous experiments.

### Cross-verification of the High Resolution Melting Analysis

**Figure 2:**
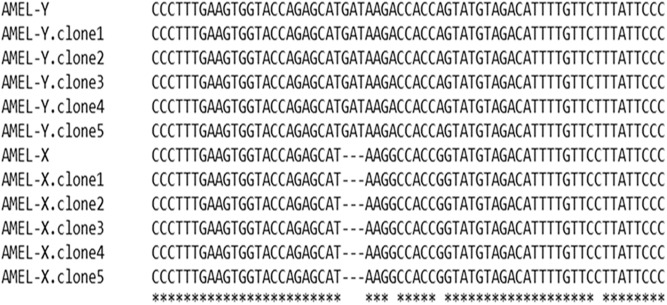
Confirmation of the HRM analysis by Sanger Sequencing. AMELX-allele and AMELY-allele fragment (61 and 64 bp, respectively) of positive controls were amplified by real-time PCR, cloned, and sequenced. The sequences obtained demonstrate the 3-bp deletion on the AMELX-allele.

The accuracy of sex determination by HRM analysis was verified by contrasting results with those obtained with other three methods. The first one was the sequencing of 5 positive clones harboring fragments from the AMELX-allele and AMELY-allele amplified from samples of known sex. The sequences obtained demonstrated the 3-bp insertion on the AMELY-allele in the male samples (Figure 2).

**Figure 3:**
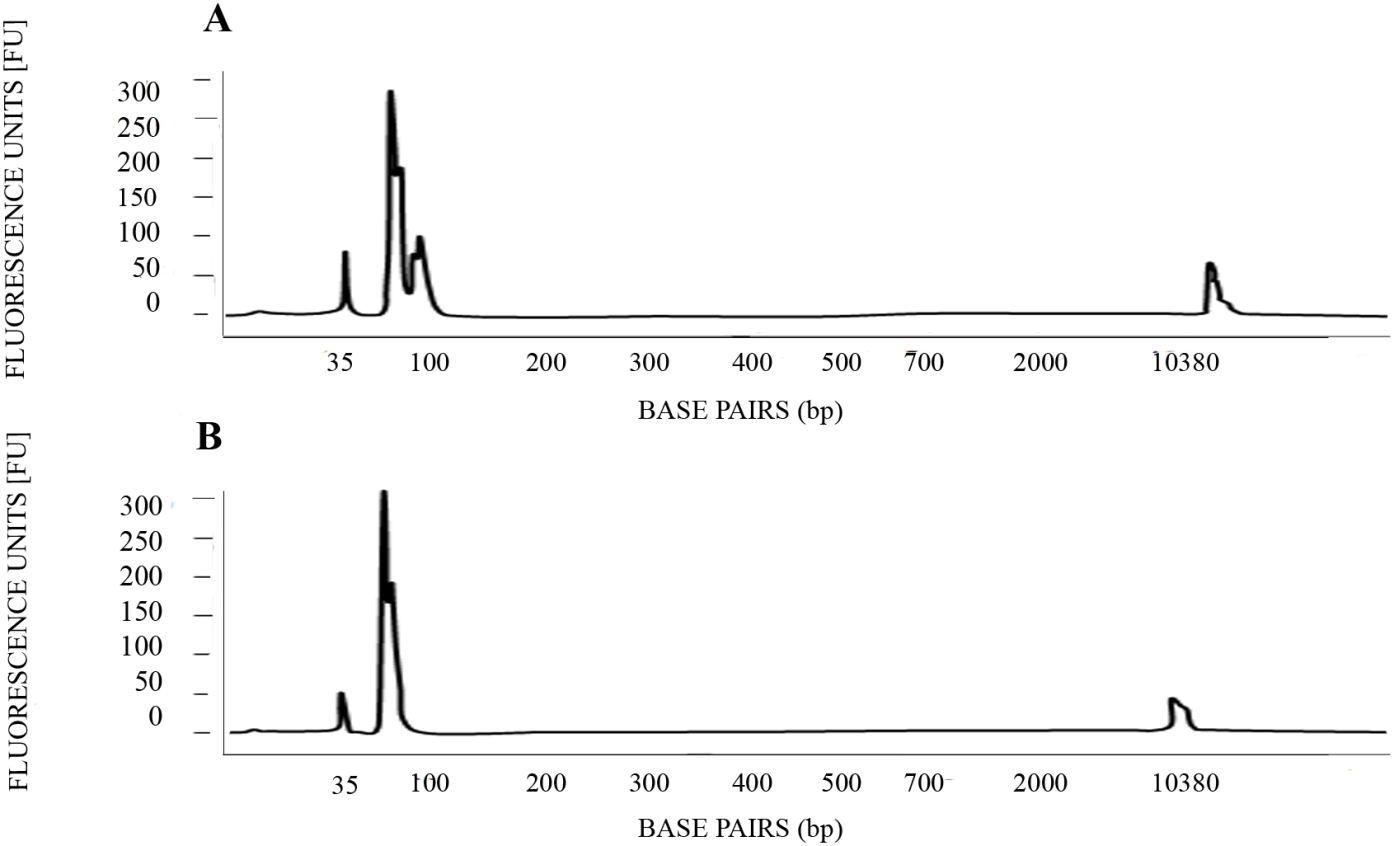
Confirmation of the HRM analysis by a Bioanalyzer assay. AMELX-allele and AMELY-allele fragment (61 and 64 bp, respectively) of positive controls were amplified by real-time PCR and then analyzed by the *Agilent 2100* *Bioanalyzer Expert High Sensitivity DNA Assay*. The results show a differential pattern in terms of amplicons length. (**A**) Male positive control. (**B**) Female positive control.

A second confirmation of the HRM analysis was conducted by a Bioanalyzer assay, in which AMELX-allele and AMELY-allele amplicons generated by Real-Time PCR from positive controls and from sex-unknown samples were analyzed. The results showed a differential pattern in terms of amplicons length between male and female individuals (Figure 3).

The third cross-verification was conducted by contrasting the HRM data with previously obtained sex determination by osteological measurements in 4 adult individuals recovered from Teopancazco, Teotihuacan (burials 2, 78, 102 and 105). The sex determination by HRM analysis matched with the sex determined by osteological measurements in all the samples analyzed.

## Discussion

Sex determination in highly degraded samples from human remains has been a challenge in the anthropological and forensic fields. Traditionally, it has been based on the visual examination of the sexual dimorphism present in the majority of human bones and dentition [1]–[45]. Nevertheless, these kinds of methods are not appropriate if the skeletons are sub-adult, incomplete or highly damaged [46]–[48]. For this reason, in the last years a large number of methods for sex determination based on molecular biology have been proposed [48]–[57]. The most widely used is based on the human amelogenin gene (AMEL) [63]–[72] because this analysis can distinguish AMEL alleles from each sexual chromosome due to genetic differences between them, as described previously by Haas-Rochholz and Weiler [73].

At the moment, different primer sets to amplify the amelogenin gene have been reported [49], [51], [52], [63], [64], [67], [68], [74]–[76]. However, the length of the fragments amplified is relatively large which causes this method to be unsuitable for heavily degraded samples. A targeted DNA fragment size of 100 bp might be too long for a successful and reliable analysis in some cases [97], [98]; especially considering this is nuclear DNA, more prone to degradation as compared to mitochondrial DNA.

In our study, the ancient samples showed a high DNA fragmentation pattern (between 40–100 bp) as assessed by a Bioanalyzer assay, indicating a general poor DNA preservation. As a result, primer sets published previously [51] were not efficient for sex determination in these samples (data not shown). This prompted us to devise a new method to sex identification analyzing smaller amplicons, suitable for heavily degraded samples. There are other published methods that involve smaller amplicons than ours, but they also involve post-PCR process, such as electrophoresis, Denaturing High Performance Liquid Chromatography (DHPLC) [78], or pyrosequencing [79], which can cause crossing-over contamination, especially in ancient samples.

Here we propose a new method based on the melting-curve analysis reported firstly by Ririe *et al.,* [80]. This method is more adequate for samples with extremely poor preservation, as it is more sensitive, while at the same time it avoids crossing-over contaminations, by not requiring any processing after real-time PCR as in other previously published methods [78], [79]. Furthermore, it allows detecting in a small amplified DNA fragment, the 3 bp deletion in the AMEL-X allele.

The use of EvaGreen in our method is relevant because it is stable both under PCR conditions and during routine storage and handling. Furthermore, no PCR interference is produced when used at low concentration, thus permitting a more robust PCR signal as well as a sharper and stronger DNA melt peak in melting-curve analyses [96], [99]. In fact, the melting-curve obtained by our method shows a clear difference in shape between male (2 peaks) and female individuals (single peak) (Figure 1).

Ancient or degraded samples are prone to the allele drop-out phenomenon, consisting in amplification failure of only one of the two alleles in a locus, mainly the largest one [100], indicating an inverse relationship between amplicon fragment and amplification failure. Therefore, a reduction of allele drop-out frequency could be expected by targeting smaller fragment sizes. On the other hand, to circumvent the problem it has been recommended to analyze several replicas of the same sample [92]. Therefore, we conducted the analysis by triplicate in all samples but one. Results from the three replicas were successfully obtained in 16 samples out of 17 samples analyzed. The exception was a sample for which DNA was finished at the second replica. However a consistent result was obtained from the two replicas for this sample.

Our method was cross-verified by three analyses. HRM results and those obtained by (i) sequencing several cloned amplicons from the AMELX-allele and AMELY-allele, (ii) a Bioanalyzer assay, and (iii) previous anthropological data [101], were all concordant.

This HRM method was efficient to determine the sex of 16 individuals of unknown sex recovered from Teopancazco, Teotihuacan. Fourteen of them were infants and three samples were from adult individuals with sex reported as no consistent by anthropological data. This illustrates the usefulness of this method to sex identification in samples in which other previously published molecular methods [51], [83] failed (see DNA preservation section above).

A variety of methodologies to sex determination have been reported previously, but are not sensitive enough on samples presenting extensive degradation of genetic material, as is the case of Mesoamerican populations subjected to warmer conditions in relation to more northern ancient populations. Our method demonstrated to be efficient, rapid and sensitive enough for sex determination in human remains showing a general poor DNA preservation. Finally, the method can be used in a high-throughput manner to analyze a relatively large number of samples in one run, at a relatively low cost. For example, including replicas and controls, 21 samples can be analyzed in a 96-well PCR plate and 84 samples in a 384-well PCR plate.

## Supporting Information

Figure S1 **DNA fragmentation pattern in a sample recovered from Teopancazco.** Main distribution of DNA fragments is in the range of 40–100 bp.
(TIF)

## References

[1] Rogers R, Saunders S (1994) Accuracy of Sex Determination Using Morphological Traits of the Human Pelvis. J Forensic Sci 39(4): 1047–1056. 8064263

[2] Kalmey JK, Rathburn TA (1996) Sex determination by discriminant function analysis of the petrous portion of the temporal bone. J Forensic Sci 41: 865–867. 8789849

[3] Burris BG, Harris EF (1998) Identification of race and sex from palate dimensions. J Forensic Sci 43: 959–963. 9729811

[4] Albanese JA (2003) Metric method for sex determination using the hipbone and the femur. J Forensic Sci 48(2): 263–273. 12664981

[5] Teke HY, Duran S, Canturk N, Canturk G (2007) Determination of gender by measuring the size of the maxillary sinuses in computerized tomography scans. Surg Radiol Anat 29(1): 9–13. 17171233 10.1007/s00276-006-0157-1

[6] Gapert R, Black S, Last J (2009) Sex determination from the foramen magnum: discriminant function analysis in an eighteenth and nineteenth century British sample. Int J Legal Med 123: 25–33. 18553095 10.1007/s00414-008-0256-0

[7] Francesquini Júnior L, Francesquini MA, De La Cruz BM, Pereira SD, Ambrosano GM, et al (2007) Identification of sex using cranial base measurements. J Forensic Odontostomatol 25(1): 7–11. 17577972

[8] May H, Peled N, Dar G, Cohen H, Abbas J, et al (2011) Hyperostosis frontalis interna: criteria for sexing and aging a skeleton. Int J Legal Med 125: 669–673. 20652712 10.1007/s00414-010-0497-6

[9] Spradley MK, Jantz RL (2011) Sex estimation in forensic anthropology: skull versus postcranial elements. J Forensic Sci 56: 289–296. 21210801 10.1111/j.1556-4029.2010.01635.x

[10] Wescott DJ, Moore-Jansen PH (2011) Metric variation in the human occipital bone: forensic anthropological applications. J Forensic Sci 46: 1159–1163. 11569559

[11] Gonzalez RA (2012) Determination of sex from juvenile crania by means of discriminant function analysis. J Forensic Sci 57: 24–34. 21939443 10.1111/j.1556-4029.2011.01920.x

[12] Krishan K, Kanchan T, Passi N, DiMaggio J (2012) Heel-Ball (HB) index-sexual dimorphism of a new index from foot dimensions? J Forensic Sci 57: 172–175. 22074354 10.1111/j.1556-4029.2011.01960.x

[13] Steyn M, Becker PJ, L’Abbé EN, Scholtz Y, Myburgh J (2012) An assessment of the repeatability of pubic and ischial measurements. Forensic Sci Int 214(1–3): 210.e1–4. 21871745 10.1016/j.forsciint.2011.07.049

[14] Uthman AT, Al-Rawi NH, Al-Timimi JF (2012) Evaluation of foramen magnum in gender determination using helical CT scanning. Dentomaxillofac Radiol 41: 197–202. 22116135 10.1259/dmfr/21276789 PMC3520293

[15] Kanchan J, Gupta A, Krishan K (2013) Estimation of sex from mastoid triangle – A craniometric analysis. J Forensic Leg Med 20(7): 855–886. 24112336 10.1016/j.jflm.2013.06.016

[16] Case DT, Ross AH (2007) Sex determination from hand and foot bone lengths. J Forensic Sci 52(2): 264–270. 17316220 10.1111/j.1556-4029.2006.00365.x

[17] Harris SM, Case DT (2012) Sexual dimorphism in the tarsal bones: implications for sex determination. J Forensic Sci 57: 295–305. 22211822 10.1111/j.1556-4029.2011.02004.x

[18] Scholtz Y, Steyn M, Pretorius E (2010) A geometric morphometric study into the sexual dimorphism of the human scapula. Homo 61(4): 253–270. 20638062 10.1016/j.jchb.2010.01.048

[19] Dabbs GR, Moore-Jansen PH (2010) A method for estimating sex using metric analysis of the scapula. J Forensic Sci 55(1): 149–152. 20002272 10.1111/j.1556-4029.2009.01232.x

[20] Berritzbeitia EL (1989) Sex determination with the head of the radius. J Forensic Sci 34: 1206–1213. 2809544

[21] Aye VO (2010) Determination of sex by arm bone dimensions. Forensic Sci Int 199(1–3): 111.e1–3. 20395082 10.1016/j.forsciint.2010.03.001

[22] Mall G, Hubig M, Buttner A, Kuznik J, Penning R, et al (2001) Sex Determination and Estimation of Stature from the Long Bones of the Arm. Forensic Sci Int 117: 23–30. 11230943 10.1016/s0379-0738(00)00445-x

[23] Purkait R (2005) Triangle identified at the proximal end of femur: a new sex determinant. Forensic Sci 147(2–3): 135–139. 10.1016/j.forsciint.2004.08.005 15567617

[24] Rissech C, Schaefer M, Malgosa A (2008) Development of the femur e implications for age and sex determination. Forensic Sci Int 180(1): 1–9. 18692331 10.1016/j.forsciint.2008.06.006

[25] Rogers TL (2009) Sex determination of adolescent skeletons using the distal humerus. Am J Phys Anthropol 140(1): 143–148. 19358295 10.1002/ajpa.21060

[26] Gonçalves D, Thompson TJU, Cunha E (2013) Osteometric sex determination of burned human skeletal remains. J Forensic Leg Med 20(7): 906–911. 24112343 10.1016/j.jflm.2013.07.003

[27] Introna F, Di Vella G, Campobasso CP (1998) Sex Determination by Discriminant Function Analysis of Patella Measurements. Forensic Sci Int 95(1): 39–45. 9718670 10.1016/s0379-0738(98)00080-2

[28] Ramadan SU, Türkmen N, Dolgun NA, Gökharman D, Menezes RG, et al (2010) Sex determination from measurements of the sternum and fourth rib using multislice computed tomography of the chest. Forensic Sci Int 197(1–3): 120.e1–5. 20083365 10.1016/j.forsciint.2009.12.049

[29] Bongiovanni R, Spradley MK (2012) Estimating sex of the human skeleton based on metrics of the sternum. Forensic Sci Int 219: 290.e1–7. 22209293 10.1016/j.forsciint.2011.11.034

[30] Kindschuh SC, Dupras TL, Cowgill LW (2010) Determination of sex from the hyoid bone. Am J Phys Anthropol 143(2): 279–284. 20853481 10.1002/ajpa.21315

[31] Rogers NL, Fluornoy LE, McCormick WF (2000) The rhomboid fossa of the clavicle as a sex and age estimator. J Forensic Sci 45(1): 61–67. 10641920

[32] Graw M, Wahl J, Ahlbrecht M (2005) Course of the meatus acusticus internus as criterion for sex differentiation. Forensic Sci Int 147: 113–117. 15567614 10.1016/j.forsciint.2004.08.006

[33] Masotti S, Succi-Leonelli E, Gualdi-Russo E (2013) Cremated human remains: is measurement of the lateral angle of the meatus acusticus internus a reliable method of sex determination? Int J Leg Med 127(5): 1039–1044. 10.1007/s00414-013-0822-y 23344564

[34] Macaluso PJ (2011) Investigation on the utility of permanent maxillary molar cusp areas for sex estimation. Forensic Sci Med Pathol 7: 233–247. 21080110 10.1007/s12024-010-9204-7

[35] Lain R, Taylor J, Croker S, Craig P, Graham J (2009) Comparative dental anatomy in disaster victim identification: lessons from the 2009 Victorian Bushfires. Forensic Sci Int 205: 36–39. 10.1016/j.forsciint.2010.06.008 20605076

[36] Ozden H, Balci Y, Demirüstü C, Turgut A, Ertugrul M (2005) Stature and sex estimate using foot and shoe dimensions. Forensic Sci Int 147: 181–184. 15567624 10.1016/j.forsciint.2004.09.072

[37] Moudgil R, Kaur R, Menezes RG, Kanchan T, Garg RK (2008) Foot index: is it a tool for sex determination? J Forensic Leg Med 15(4): 223–226. 18423354 10.1016/j.jflm.2007.10.003

[38] Zeybek G, Ergur I, Demiroglu Z (2008) Stature and gender estimation using foot measurements. Forensic Sci Int 181(1–3): 54.e1–5. 18829191 10.1016/j.forsciint.2008.08.003

[39] Atamturk D (2010) Estimation of sex from the dimensions of foot, footprints, and shoe. Anthropol Anz 68(1): 21–29. 20954453 10.1127/0003-5548/2010/0026

[40] Jowaheer V, Agnihotri AK (2011) Sex identification on the basis of hand and foot measurements in Indo-Mauritian population–a model based approach. J Forensic Leg Med 18(4): 173–176. 21550567 10.1016/j.jflm.2011.02.007

[41] Kanchan T, Krishan K (2011) Anthropometry of hand in sex determination of dismembered remains - a review of literature. J Forensic Leg Med 18: 14–17. 21216373 10.1016/j.jflm.2010.11.013

[42] Kanchan T, Kumar GP, Menezes RG (2008) Index and ring finger ratio - a new sex determinant in south Indian population. Forensic Sci Int 181(1–3): 53.e1–4. 18814978 10.1016/j.forsciint.2008.08.002

[43] Kanchan T, Kumar GP (2010) Index and ring finger ratio - a morphologic sex determinant in South-Indian children. Forensic Sci Med Pathol 6: 255–260. 20369311 10.1007/s12024-010-9156-y

[44] Kanchan T, Kumar GP, Menezes RG, Rastogi P, Rao PP, et al (2010) Sexual dimorphism of the index to ring finger ratio in South Indian adolescents. J Forensic Leg Med 17(5): 24–36. 10.1016/j.jflm.2010.02.009 20569949

[45] Krishan K, Kanchan T, Asha N, Kaur S, Chatterjee PM, et al (2013) Estimation of sex from index and ring finger in a North Indian population. J Forensic Leg Med 20(5): 471–479. 23756517 10.1016/j.jflm.2013.03.004

[46] Rodríguez JV (1994) Introducción a la Antropología forense. Análisis e identificación de restos óseos humanos. Anaconda ed. Bogotá, Colombia.139 p.

[47] Schmidt D, Hummel S, Herrmann B (2003) Brief Communication: Multiplex X/Y-PCR Improves Sex Identification in aDNA Analysis. Am J Phys Anthropol 121(4): 337–341. 12884315 10.1002/ajpa.10172

[48] Daskalaki E, Anderung C, Humphrey L, Götherström A (2011) Further developments in molecular sex assignment: a blind test of 18th and 19th century human skeletons. J Archaeol Sci 38(6): 1326–1330.

[49] Akane A, Shiono H, Matsubara K, Nakahori Y, Seki S, et al (1991) Sex identification of forensic specimens by polymerase chain reaction (PCR): two alternative methods. Forensic Sci Int 49(1): 81–88. 2032670 10.1016/0379-0738(91)90174-h

[50] Hummel S, Herrmann B (1991) Y-Chromosome-Specific DNA amplified in ancient human bone. Naturwissenschaften 78: 266–267. 1922388 10.1007/BF01134353

[51] Sullivan KM, Mannucci A, Kimpton CP, Gill P (1993) A rapid and quantitative DNA sextest: fluorescence-based PCR analysis of X-Y homologous gene amelogenin. Biotechniques. 15: 636–641. 8251166

[52] Faerman M, Filon D, Kahila G, Greenblatt C, Smith P, et al (1995) Sex identification of archaeological human remains based on amplification of the X and Y amelogenin alleles. Gene 167(1–2): 327–332. 8566801 10.1016/0378-1119(95)00697-4

[53] Stone AC, Milner GR, Pääbo S, Stoneking M (1996) Sex determination of ancient human skeletons using DNA. Am J Phys Anthropol 99: 231–238. 8967324 10.1002/(SICI)1096-8644(199602)99:2<231::AID-AJPA1>3.0.CO;2-1

[54] Götherström A, Liden K, Ahlstrom T, Kallersjo M, Brown TA (1997) Osteology, DNA and sex identification: morphological and molecular sex identifications of five neolithic individuals from Ajvide, Gotland. Int J Osteoarchaeol 7: 71–81.

[55] Lassen C, Hummel S, Herrmann B (2000) Molecular sex identification of stillborn and neonate individuals (“Traufkinder”) from the burial site Aegerten. Anthropol Anz 58(1): 1–8. 10816779

[56] Meyer E, Wiese M, Bruchhaus H, Claussen M, Klein A (2000) Extraction and amplification of authentic DNA from ancient human Remains. Forensic Sci Int 113: 87–90. 10978606 10.1016/s0379-0738(00)00220-6

[57] Gibbon V, Paximadis M, Strkalj G, Ruff P, Penny C (2009) Novel methods of molecular sex identification from skeletal tissue using the amelogenin gene. Forensic Sci Int Genet 3: 74–79. 19215875 10.1016/j.fsigen.2008.10.007

[58] Pilli E, Modi A, Serpico C, Achilli A, Lancioni H, et al (2013) Monitoring DNA contamination in handled vs. directly excavated ancient human skeletal remains. PLoS One 8(1): e52524. 23372650 10.1371/journal.pone.0052524 PMC3556025

[59] Skaletsky H, Kuroda-Kawaguchi T, Minx PJ, Cordum HS, Hillier L, et al (2003) The male-specific region of the human Y chromosome is a mosaic of discrete sequence classes. Nature 423: 825–837. 12815422 10.1038/nature01722

[60] Jobling MA, Lo IC, Turner DJ, Bowden GR, Lee AC, et al (2007) Structural variation on the short arm of the human Y chromosome: recurrent multigene deletions encompassing amelogenin Y. Hum Mol Genet. 16(3): 307–316. 17189292 10.1093/hmg/ddl465 PMC2590852

[61] Tozzo P, Giuliodori A, Corato S, Ponzano E, Rodriguez D, et al (2013) Deletion of amelogenin Y-locus in forensics: Literature revision and description of a novel method for sex confirmation. J Forensic Leg Med 20(5): 387–391 doi:10.1016/j.jflm.2013.03.012 23756502 10.1016/j.jflm.2013.03.012

[62] Skoglund P, Storå J, Götherström A, Jakobsson M (2013) Accurate sex identification of ancient human remains using DNA shotgun sequencing. J Archaeol Sci 40(12): 4477–4482 doi:10.1016/j.jas.2013.07.004

[63] Nakahori Y, Hamano K, Iwaya M, Nakagome Y (1991) Sex identification by polymerase chain reaction using X-Y homologous primer. Am J Med Genet 39(4): 472–473. 1877627 10.1002/ajmg.1320390420

[64] Nakahori Y, Takenaka O, Nakagome Y (1991) A human X-Y homologous region encodes amelogenin. Genomics 9: 264–269. 2004775 10.1016/0888-7543(91)90251-9

[65] Lau EC, Mohandas TK, Shapiro LJ, Slavkin HC, Snead ML (1989) Human and mouse amelogenin loci are the sex chromosomes. Genomics 4: 162–168. 2737677 10.1016/0888-7543(89)90295-4

[66] Lau EC, Slavkin HC, Snead M (1990) Analysis of human enamel genes. Insights into genetic disorders of enamel. Cleft Palate J 27(2): 121–130. 2187631 10.1597/1545-1569(1990)027<0121:aohegi>2.3.co;2

[67] Akane A, Seki S, Shiono H, Nakamura H, Hasegawa M, et al (1992) Sex determination on Forensic Samples by dual PCR amplification of an X-Y homologous gene. Forensic Sci Int 52(2): 143–148. 1601346 10.1016/0379-0738(92)90102-3

[68] Bailey D, Affara N, Ferguson-Smith M (1992) The X-Y homologous gene amelogenin maps to the short arms of both the X and Y chromosomes and is highly conserved in primates. Genomics 14: 203–205. 1427830 10.1016/s0888-7543(05)80310-6

[69] Salido EC, Yen PH, Koprivnikar K, Yu LC, Shapiro LJ (1992) The human enamel protein gene amelogenin is expressed from both the X and the Y chromosomes. Am J Hum Genet 50: 303–316. 1734713 PMC1682460

[70] Sasaki S, Shimokawa H (1995) The amelogenin gene. Int J Dev Biol 39: 127–133. 7626398

[71] Slavkin H (1997) Sex, enamel and forensic dentistry: A search for identity. J Am Dent Assoc 128(7): 1021–1025. 9231609 10.14219/jada.archive.1997.0311

[72] Zemoskova E, Frolova S, Sleptsova Z, Ivanov P (2003) Study of species specificity of the amelogenin system for genetic sex determination. Sud Med Ekspert 46(4): 19–22. 12939838

[73] Haas-Rochholz H, Weiler G (1997) Additional primer sets for an amelogenin gene PCR-based DNA-sex test. Int J Legal Med 110: 312–315. 9387013 10.1007/s004140050094

[74] Mannucci A, Sullivan KM, Ivanov PL, Gill P (1994) Forensic application of a rapid andquantitative DNA sex test by amplification of the X-Y homologous gene amelogenin. Int J Legal Med 106: 190–193. 8038111 10.1007/BF01371335

[75] Lassen C, Hummel S, Herrmann B (1996) PCR based sex identification of ancient human bones by amplification of X- and Y-chromosomal sequences: a comparisson. Ancient Biomol 1: 25–33.

[76] Velarde-Félix JS, Molina-Benítez CE, Solórzano-Rosales S, Cázarez-Salazar SG, Rendón-Aguilar H, et al (2008) Identificación del sexo mediante análisis molecular del gen de la amelogenina. Rev Mex Patol Clin 55(1): 17–20.

[77] Taberlet P, Friffin S, Goossens B, Questiau S, Manceau V, et al (1996) Reliable genotyping of samples with very low DNA quantities using PCR. Nucleic Acids Res 24: 3189–3194. 8774899 10.1093/nar/24.16.3189 PMC146079

[78] Shinka T, Naroda T, Tamura T, Sasahara K, Nakahori Y (2001) A rapid and simple method for sex identification by heteroduplex analysis, using denaturing high-performance liquid chromatography (DHPLC). J Hum Genet 46: 263–266. 11355016 10.1007/s100380170076

[79] Tschentscher F, Frey U, Bajanowski T (2008) Amelogenin sex determination by pyrosequencing of short PCR products. Int J Legal Med 122: 333–335. 18351373 10.1007/s00414-008-0228-4

[80] Ririe KM, Rasmussen RP, Wittwer CT (1997) Product differentiation by analysis of DNA melting curves during the polymerase chain reaction. Anal Biochem 245: 154–160. 9056205 10.1006/abio.1996.9916

[81] Wittwer CT, Herrmann MG, Moss AA, Rasmussen RP (1997) Continuous fluorescence monitoring of rapid cycle DN amplification. Biotechniques 22: 130–131. 8994660 10.2144/97221bi01

[82] Wittwer CT, Ririe KM, Andrew RV, David DA, Gundry RA, et al (1997) The LightCycler: a microvolume multisample fluorimeter with rapid temperature control. Biotechniques 22: 176–181. 8994665 10.2144/97221pf02

[83] Andréasson H, Allen M (2003) Rapid quantification and sex determination of forensic evidence materials. J Forensic Sci 48: 1280–1287. 14640271

[84] Karsai A, Muller S, Platz S, Hauser MT (2002) Evaluation of a homemade SYBR green I reaction mixture for real-time PCR quantification of gene expression. Biotechniques 32(4): 790–792. 11962601 10.2144/02324st05 PMC4353838

[85] Alonso A, Martín P, Albarrán C, García P, Primorac D, et al (2003) Specific quantification of human genomes from low copy number DNA samples in forensic and ancient DNA studies. Croat Med J 44(3): 273–280. 12808718

[86] Giglio S, Monis PT, Saint CP (2003) Demostration of preferential binding of SYBR Green I to specific DNA fragments in real-time multiplex PCR. Nucleic Acids Res 31(22): 136. 10.1093/nar/gng135 PMC275573 14602929

[87] Varga A, James D (2006) Real-time RT-PCR and SYBR Green I melting curve analysis for the identification of Plum pox virus strains C, EA, and W: effect of amplicon size, melt rate, and dye translocation. J Virol Methods 132(1–2): 146–153. 16293321 10.1016/j.jviromet.2005.10.004

[88] Taberlet P, Friffin S, Goossens B, Questiau S, Manceau V, et al (1996) Reliable genotyping of samples with very low DNA quantities using PCR. Nucleic Acids Res 24: 3189–3194. 8774899 10.1093/nar/24.16.3189 PMC146079

[89] Gagneux P, Boesch C, Woodruff DS (1997) Microsatellite scoring errors associated with noninvasive genotyping based on nuclear DNA amplified from shed hair. Mol Ecol 6: 861–868. 9301074 10.1111/j.1365-294x.1997.tb00140.x

[90] Santos FR, Pandya A, Tyler-Smith C (1998) Reliability of DNA-based sex tests. Nat Genet 18: 103. 9462733 10.1038/ng0298-103

[91] Lattanzi W, Di Giacomo MC, Lenato GM, Chimienti G, Voglino G, et al (2005) A large interstitial deletion encompas- sing the amelogenin gene on the short arm of the Y chromosome. Hum Genet 116: 395–401. 15726419 10.1007/s00439-004-1238-z

[92] Kim KY, Kwon Y, Bazarragchaa M, Park AJ, Bang H, et al (2013) A real-time PCR-based amelogenin Y allele dropout assessment model in gender typing of degraded DNA samples. Int J Legal Med 127(1): 55–61. 22237796 10.1007/s00414-011-0663-5

[93] Steinlechner M, Berger B, Niederstätter H, Parson W (2002) Rare failures in the amelogenin sex test. Int J Legal Med 116(2): 117–120. 12056519 10.1007/s00414-001-0264-9

[94] Thangaraj K, Reddy AG, Singh L (2002) Is the amelogenin reliable for gender identification in forensic casework and prenatal diagnosis? Int J Leg Med 116: 121–123. 10.1007/s00414-001-0262-y 12056520

[95] Chang YM, Burgoyne LA, Both K (2003) Higher failures of amelogenin sex test in an Indian population group. J Forensic Sci 48: 1309–1313. 14640276

[96] Montiel R, Malgosa A, Francalacci P (2001) Authenticating Ancient Human Mitochondrial DNA. Hum Biol 73(5): 689–713. 11758690 10.1353/hub.2001.0069

[97] Malmström H, Svensson EM, Gilbert MT, Willerslev E, Götherström A, et al (2007) More on Contamination: The use of asymmetric molecular behavior to identify authentic ancient human DNA. Mol Biol Evol 24(4): 998–1004. 17255122 10.1093/molbev/msm015

[98] Green RE, Briggs AW, Krause J, Prüfer K, Burbano HA, et al (2009) The Neandertal genome and ancient DNA authenticity. EMBO J 28(17): 2494–502. 19661919 10.1038/emboj.2009.222 PMC2725275

[99] Herrmann MG, Durtschi JD, Bromley LK, Wittwer CT, Voelkerding KV (2006) Amplicon DNA Melting Analysis for Mutation Scanning and Genotyping: Cross-Platform Comparison of Instruments and dyes. Clin Chem 53: 494–503. 10.1373/clinchem.2005.063438 16423901

[100] Tvedebrink T, Eriksen PS, Mogensen HS, Morling N (2012) Statistical model for degraded DNA samples and adjusted probabilities for allelic drop-out. Forensic Sci Int 6(1): 97–101. 10.1016/j.fsigen.2011.03.001 21458395

[101] Manzanilla LR (2012) Banco de datos del sitio Teopancazco. Proyecto “Teotihuacan: elite y gobierno”, 1997–2005. In: Manzanilla LR, editor. *Estudios arqueométricos del centro de barrio de Teopancazco en Teotihuacan*, UNAM, México. pp. 467–552.

