# Supplementary figures and images for: Sex Determination in Highly Fragmented Human DNA by High-Resolution Melting (HRM) Analysis
Source: PLoS One. 2014 Aug 6;9(8):e104629. doi: 10.1371/journal.pone.0104629 (PMC4123986; doi:10.1371/journal.pone.0104629)

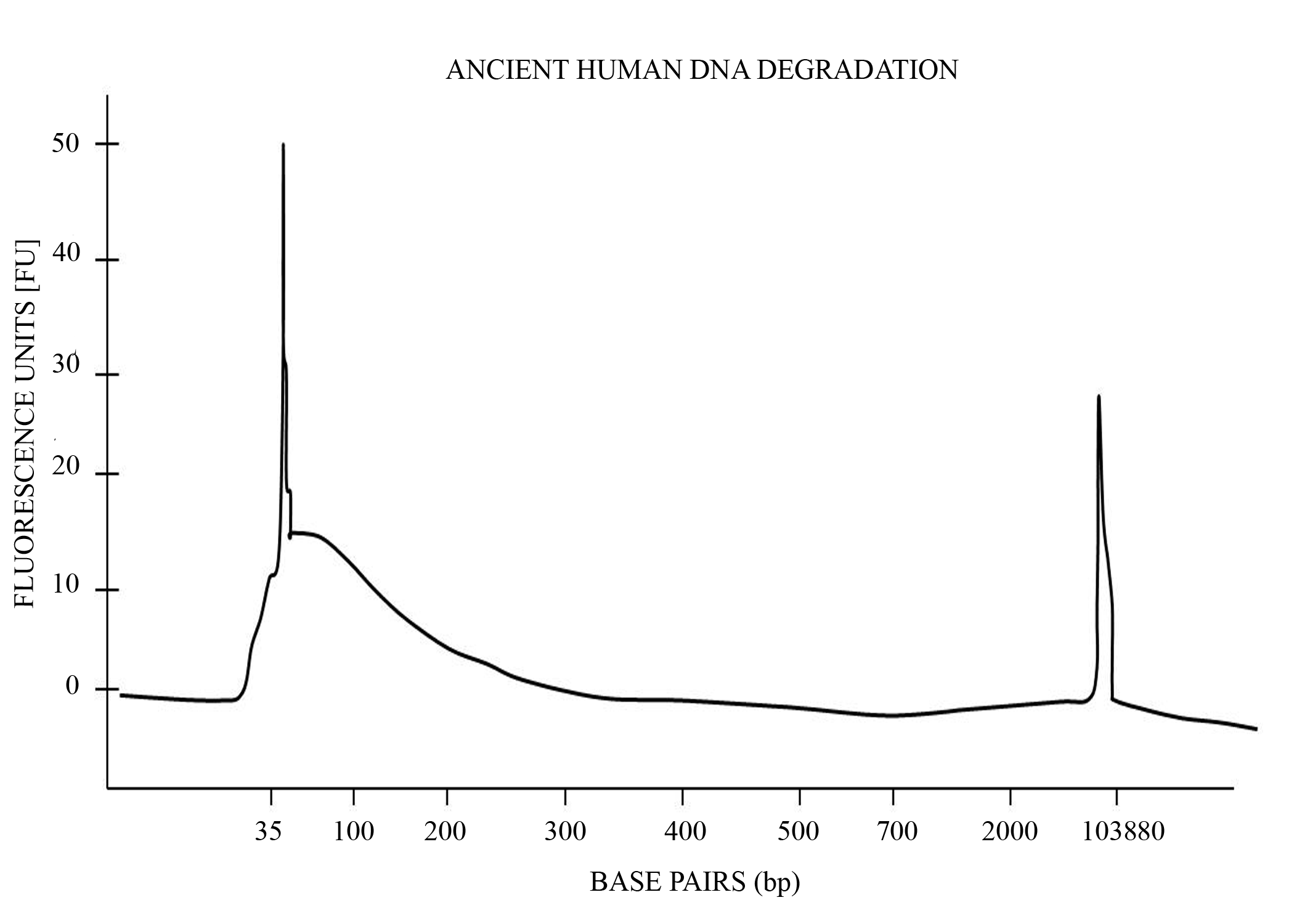

Supplement: Figure S1 — DNA fragmentation pattern in a sample recovered from Teopancazco. Main distribution of DNA fragments is in the range of 40–100 bp. (TIF) [file pone.0104629.s001.tif]
